# Overrepresentation of unaccompanied refugee minors in inpatient psychiatric care

**DOI:** 10.1186/s40064-015-0902-1

**Published:** 2015-03-15

**Authors:** Björn Ramel, Jakob Täljemark, Anna Lindgren, Björn Axel Johansson

**Affiliations:** Office for Healthcare “Sund”, Child & Adolescent Psychiatry, SE-205 02 Malmö, Sweden; Department of Clinical Sciences Lund, Division of Child & Adolescent Psychiatry, Lund University, SE-221 85 Lund, Sweden; Office for Healthcare “Sund”, Child & Adolescent Psychiatry, SE-221 85 Lund, Sweden; Department of Mathematical Statistics, Centre for Mathematical Sciences, Lund University, SE-223 62 Lund, Sweden; Department of Health Sciences, Clinical Health Promotion Centre, Lund University, SE-205 02 Malmö, Sweden; Office for Healthcare “Sund”, Child & Adolescent Psychiatry, Regional Inpatient Care, Emergency Unit, SE-205 02 Malmö, Sweden

**Keywords:** Unaccompanied refugee minors, Child & Adolescent Psychiatry, Inpatient care, Involuntary care

## Abstract

**Background:**

Unaccompanied refugee minors (URMs) have high levels of psychiatric symptoms, and concerns for their access to mental health services have been raised. From the mid-2000s, an increasing number of asylum-seeking URMs, mainly adolescent boys from Afghanistan, have been referred to the Child & Adolescent Psychiatry emergency unit in Malmö, Sweden. The aim of the study was to compare inpatient psychiatric care between URMs and non-URMs.

**Findings:**

All admissions in 2011 at the emergency unit were identified and divided into URMs (n = 56) and non-URMs (n = 205). On the basis of unique patients’ first treatment occasion, a group level analysis was performed on gender, age, treatment duration, additional treatment occasions/patient, involuntary care, involuntary care by gender, and ICD-10 principal diagnosis. To retrieve further sample characteristics, a questionnaire was administered to the physicians responsible for admitting patients in 2011.

More URMs than non-URMs exhibited self-harm or suicidal behaviour in conjunction with referral. 86% of URMs were admitted with symptoms relating to stress in the asylum process. In the catchment area, 3.40% of the URM population received inpatient care and 0.67% inpatient involuntary care, compared to 0.26% and 0.02% respectively of the non-URM population, both comparisons p < 0.001. There were more boys in the URM group (95%) compared to the non-URM group (29%). A difference in use of involuntary care disappeared after adjusting for gender. No differences were found in diagnoses except for neurotic disorders (F40-48), which were more common in the URM group.

**Conclusion:**

From an epidemiological perspective, URMs were overrepresented in inpatient psychiatric care.

## Introduction

Unaccompanied refugee minors (URMs) are a diverse group with loss and vulnerability as common denominators. URM is defined as any child under the age of 18 who is physically separated from both parents and is an asylum seeker, recognised refugee or other displaced person (Bean et al. 2007). Compared to accompanied refugee children and non-immigrants, URMs have more traumatic stress reactions (Bean et al. 2007; Hodes et al. 2008). The prevalence of psychiatric disorders has been found to be higher in URMs compared to refugee minors accompanied by families (Wiese & Burhorst 2007). A Belgian study reported severe symptoms of anxiety, depression and PTSD in 37-47% of 166 URMs (Derluyn & Broekaert 2007). A third of 222 male Afghan asylum-seeking URMs aged 13–18 years in the UK reported above threshold for anxiety, emotional and behavioural problems and PTSD, and 23% for depression (Bronstein et al. 2012; Bronstein et al. 2013). In a Norwegian study structured diagnostic interviews with 160 male asylum-seeking URMs aged 14–20 years showed that 41.9% had a psychiatric disorder, PTSD being the most prevalent (30.6%), followed by depression (9.4%), agoraphobia (4.4%) and GAD (3,8%) (Jakobsen et al. 2014).

The corresponding prevalences for minors in general are also heterogeneous but lower; anxiety 5-9% (Klein 1994), depression 2–8% (Harrington 1994; Thapar et al. 2012) and PTSD 4% (Katzman et al. 2014).

The United Nations High Commissioner for Refugees (UNHCR) estimates that 17,700 unaccompanied children lodged asylum applications in 69 countries in 2011, including 13,300 in Europe (United Nations High Commissioner for Refugees (UNHCR) 2012). Sweden registered the highest number of applications in Europe, about 2,700, after years of steady increase (2005: 400, 2008: 1,500) (United Nations High Commissioner for Refugees (UNHCR) 2012; Swedish Migration Board 2012). The majority of URMs arriving in Sweden in 2011 were boys (85%), aged between 13 and 17 years (92%) and originated from Afghanistan (64%) (Swedish Migration Board 2012). Reflecting this trend, from the mid-2000s an increasing number of URMs have been referred to the Child & Adolescent Psychiatry emergency unit in Malmö, Sweden. Concerns for unmet mental health needs among URMs have been expressed (Bean et al. 2006; Michelson & Sclare 2009).

Research on young refugees’ utilisation of mental health services is limited, particularly regarding inpatient psychiatric services and involuntary care. Bhui et al. reported that refugees were not overrepresented in inpatient psychiatric care compared to non-refugees. However, a separate analysis on URMs was not included (Bhui et al. 2006).

## Aim

The aim of this study was to compare inpatient psychiatric care between URMs and non-URMs.

## Sample

In 2011, 253,000 children and adolescents resided in the catchment area of the emergency unit, Skåne County. In the age group 12–17 years the number was 81,077 (D. Nilsson, Skåne Regional Council, personal communication). Approximately 500 of these were URMs with permanent residency, while another 1,157 applied for asylum at the Swedish Migration Board office in Malmö, some of which were transferred to a destination outside the catchment area within a few days (L. Jönsson, County Administrative Board of Skåne, personal communication). The Migration board office in Malmö is the only one in the catchment area. We estimate that URMs, at most, accounted for 2% of the catchment population aged 12 to 17 years in 2011. 411 accompanied minors aged 12–17 years applied for asylum in Malmö 2011 (L. Jönsson, County Administrative Board of Skåne, personal communication). Based on the authors’ clinical experience, it is assessed that very few, if any, accompanied refugee minors were admitted to the emergency unit in 2011.

In Sweden asylum-seeking children have the same rights to health care as other children. Outpatient mental health services is offered by general Child & Adolescent psychiatric care units situated in 11 towns across the catchment area. Physicians, psychologists, counsellors, nurses and care workers staff these units. Interpreters are used when needed, both in out- and inpatient settings.

The clinical sample comprised all unique inpatients (n = 261) admitted in 2011, including 56 URMs and 205 non-URMs. The reason for not including a larger database was twofold: a specific code for URMs in the administrative files did not exist before 2011. Second, the emergency unit got a larger geographic assignment in 2010, i.e. it would be difficult to compare 2011 with earlier years.

In the sample about 75% of URMs were asylum-seeking minors from Afghanistan. Many URMs arrived in Sweden via other European countries and reported abuse or maltreatment in some of these countries, in addition to stressful life events in their home country. Prior to referral, a majority had received briefings or decisions in the asylum process indicating that they should return to one of these European countries, under the terms of the Dublin Regulation (Council Regulation [EC] No 343/2003) of the EU. The regulation stipulates that the member state where the asylum seeker was first registered in the EU is responsible for assessing the asylum application.

URMs often came to the emergency unit accompanied by and on initiative from housing staff. Less common sources of referrals were the legal guardian, an outpatient Child & Adolescent mental health care unit, a GP, social authorities, the school or the Paediatric emergency unit. Depending on circumstances, the actual transport to the emergency unit was sometimes carried out by the police or ambulance. Non-URMs normally arrived to the emergency unit accompanied by their caregivers. Apart from this, the sources of referral did not differ between URMs and non-URMs.

## Methods

Since 1^st^ of January 2011 URMs have been assigned a specific code in the administrative files at the Child & Adolescent Psychiatry department in Malmö. The coding was manually done by secretaries according to information in the patient’s medical record. Using this code, all admissions in 2011 were divided into URMs and non-URMs. The specific URM-code excludes any accompanied refugee minors in the analysis of the URM group.

Selecting unique patients’ first treatment occasion, a group-level analysis was performed on gender, age, treatment duration, additional treatment occasions/patient, involuntary care, involuntary care by gender, and ICD-10 principal diagnosis divided into nine clusters. Using each patients’ unique identification number multiple admissions could be identified in the administrative files. In Sweden asylum-seeking individuals are also given a unique identification number, though it is different from those with residency or Swedish citizenship. The rationale for basing the analysis on unique patients’ first treatment occasion was to reduce sample heterogeneity.

An epidemiological comparison regarding voluntary and involuntary inpatient care used the catchment population aged 12–17 years as reference.

The administrative files did not allow distinction between asylum-seeking URMs and URMs with residency, and the study design did not permit access to medical records of individual patients. From clinical experience the authors estimate that at least 95% of inpatient URMs were asylum seekers. One author, BAJ, worked full time at the emergency unit in 2011 and attended over 90% of the morning reports where all inpatients were discussed and the doctors on duty reported admitted patients. The authors BR and JT were regularly on-call at the emergency unit. Since the asylum process is a central issue for URMs, it was brought up in morning reports, and in the daily patient assessments, which the legal guardian and/or housing staff often attended.

In a questionnaire the 48 physicians responsible for admitting patients during office and non-office hours in 2011 were asked to estimate the share of admitted patients that displayed self-harm or suicidal behaviour (URMs and non-URMs) and stress relating to the asylum process (URMs) (Table 1). The questionnaire was administered in spring 2014. After two email reminders and one phone call 37 physicians (77%) completed the questionnaire. Of the 11 excluded, four reported that they could not recall the requested circumstances, one answered only two out of three questions, and six did not reply. The vast majority of on-call physicians did also in periods work at the emergency unit during office hours.

**Table 1:** **Questions administered to 48 physicians responsible for admitting patients during office and non-office hours in 2011 at the Child & Adolescent Psychiatry emergency unit in Malmö, Sweden**

1.	How many of the URMs that you admitted during 2011 were admitted for reasons relating to stress in the asylum process, e.g. rejection of application, prolonged process or anxiety ahead of decision?
2.	How many of the URMs that you admitted during 2011 exhibited self-harm or suicidal behaviour in conjunction with admittance?
3.	How many of the non-URMs that you admitted during 2011 exhibited self-harm or suicidal behaviour in conjunction with admittance?

## Statistics

All tests of differences in proportions were performed using Fisher’s exact test. The p-values in the post hoc comparisons of diagnoses were adjusted for nine multiple comparisons using the Bonferroni correction. Comparisons of treatment duration and age were performed using the Mann–Whitney *U*-test. The difference in proportions of self-harm or suicidal behaviour in conjunction with referral was tested using paired *t*-test in order to take into account possible systematic differences between physicians’ estimations. The physicians were asked two questions similar for all patients. P-value < 0.05 was considered significant, except when using the Bonferroni correction where P-value < 0.05/9 = 0.006 was used.

## Results

In the catchment area, 3.40% (56/1,657) of the URM population received inpatient care and 0.67% involuntary inpatient care, compared to 0.26% (205/81,077) and 0.02% respectively of the non-URM population. Both comparisons were significant (p < 0.001).

Physicians estimated on average that a mean (SD) of 76% (21.8) of admitted URMs and 58% (21.1) of non-URMs respectively exhibited self-harm or suicidal behaviour in conjunction with referral (p <0.001). 86% (13.2) of URMs were admitted with symptoms related to stress in the asylum process.

There were more boys in the URM group compared to the non-URMs, 95% and 29% respectively (Table 2). The URMs were older.

**Table 2:** **Unique patients’ first treatment episode 2011: treatment occasions, gender, age, treatment duration, additional treatment occasions/patient, involuntary care, involuntary care by gender and ICD-10 principal diagnostic clusters in URMs and non-URMs respectively at the Child & Adolescent Psychiatry emergency unit in Malmö, Sweden**

**Variables**	**All patients**	**URM**	**non-URM**	**URM vs. non-URM**
	**N = 261**	**N = 56**	**N = 205**	**p-values**
Treatment occasions	372	86	286	-
Gender	F 149 (57.1%)	F 3 (5.4%)	F 146 (71.2%)	p < 0.001
	M 112 (42.9%)	M 53 (94.6%)	M 59 (28.8%)	
Age, years (SD)	15.1 (1.8)	15.9 (1.5)	14.9 (1.8)	p < 0.001
Treatment duration, days (SD)	8.7 (16.6)	6.2 (8.6)	9.4 (18.1)	ns
Additional treatment occasions/patient	0.43	0.54	0.40	ns
Involuntary care	28 (10.7%)	11 (19.6%)	17 (8.3%)	p = 0.026
Involuntary care by gender	F 8 (5.4%)	F 0 (0.0%)	F 8 (5.5%)	ns
	M 20 (17.9%)	M 11 (20.8%)	M 9 (15.3%)	
Neurotic, stress-related and somatoform disorders (F40-F48)	106 (40.6%)	43 (76.8%)	63 (30.7%)	p < 0.001
Mood disorders (F30-F39)	74 (28.4%)	9 (16.1%)	65 (31.7%)	ns
Behavioural and emotional disorders (F90-F98)	28 (10.7%)	1 (1.8%)	27 (13.2%)	ns
Behavioural syndromes associated with physiological disturbances and physical factors (F50-F59)	13 (5.0%)	0 (0.0%)	13 (6.3%)	ns
Mental and behavioural disorders due to psychoactive substance use (F10-F19)	13 (5.0%)	2 (3.6%)	11 (5.4%)	ns
Disorders of psychological development (F80-F89)	5 (1.9%)	0 (0.0%)	5 (2.4%)	ns
Schizophrenia, schizotypal, and delusional disorders (F20-F29)	3 (1.1%)	0 (0.0%)	3 (1.5%)	ns
Mental retardation (F70-F79)	2 (0.8%)	0 (0.0%)	2 (1.0%)	ns
Symptoms and signs (R00-R99) & Factors influencing health status (Z00-Z99)	17 (6.5%)	1 (1.8%)	16 (7.8%)	ns

Standard deviation or percentage in brackets. F = Female, M = Male.

As inpatients, URMs comprised 21% of unique patients’ first treatment occasion (56/261), and 39% of involuntary care (11/28). The URM group received more involuntary care compared to non-URMs, but the difference disappeared after adjusting for gender. There were no differences in diagnoses except for neurotic disorders (F40-48), which were more common in the URM compared to the non-URM group (Table 2).

## Discussion

The major finding was that URMs, from an epidemiological perspective, were markedly overrepresented in both voluntary and involuntary inpatient psychiatric care. URMs in different host countries and with different origins have high levels of mental health problems, including PTSD, depression, anxiety and emotional and behavioural problems (Huemer et al. 2009; Bronstein et al. 2012; Bronstein et al. 2013; Jakobsen et al. 2014). The psychiatric morbidity, along with the higher rate of self-harm or suicidal behaviour, contributed to the overrepresentation of URMs in voluntary and involuntary inpatient psychiatric care (Gordon 2002).

Another possible explanation is lack of outpatient mental health services adjusted for the URM population. Bean et al. found that almost 50% of URMs reported an unmet mental health need, and that URMs with a need experienced higher levels of emotional distress than non-URMs (Bean et al. 2006). This is in line with our finding that URMs exhibited more self-harm or suicidal behaviour than non-URMs. In the catchment area there is no mental health unit specifically targeted for URMs, but one unit, The Unit for War and Torture Victims, serves children and adolescents with complex posttraumatic symptomatology. Recently, the unit initiated a training programme for staff at the supervised homes for URMs. The teaching has focused on psychotraumatology and the importance of coordinating support measures involving school, legal guardians and social services.

The overrepresentation may also be related to the insecure asylum status of URMs. This uncertainty, including difficulties obtaining legal residence documents, is associated with psychological problems (Fazel et al. 2012; Vervliet et al. 2014). At referral, 86% of URMs displayed symptoms relating to stress in the asylum process, including indications or a decision from migration authorities that would involve returning to another EU country often associated with previous adversities. This seems to have created a sense of despair and elicited an acute stress reaction (Hodes et al. 2008; Bronstein et al. 2013). Higher rate of suicide attempts and hospital-treated suicidal behaviour in groups of asylum seekers have been documented (Staehr & Munk-Andersen 2006; Goosen et al. 2011). Further, immigrant background has been associated with use of restraint among adolescents in psychiatric treatment (Furre et al. 2014).

The URM group was older and contained more boys compared to the non-URM group. This picture probably reflects a similar gender and age difference in the catchment population. This is not surprising considering that younger refugee children and particularly girls may be exposed to more threats than older boys during a strenuous journey from a country like Afghanistan to Sweden without caregivers. The epidemiological overrepresentation of URMs in both inpatient and involuntary care may partly be related to age (Pottick et al. 1995). From an inpatient perspective, the difference in involuntary care between the two groups is too large to be explained by age only. The difference was instead explained by gender, i.e. in both groups boys were more often treated involuntarily than girls. Male gender has been associated with increased use of coercive measures among adolescents, though results are diverging (Ulla et al. 2012).

In Sweden the legal framework for treating patients involuntarily is the same at all ages. While the capacity to refuse inpatient treatment is compromised for younger patients, there is no inherent difference between URMs and non-URMs in this regard. There is legal precedence in Sweden that children from 12 years have mandate to deny health care, meaning that involuntary care should be applied if necessary, rather than having the parents decide for the child.

In the catchment area there were accompanied refugee minors seeking asylum. Very few, if any, were admitted for inpatient care. One reason for this, besides the limited number in the catchment area, was probably the presence of caregivers.

The higher frequency of cluster F40-48 diagnoses in the URMs compared to the non-URM group was probably more a result of acute stress reactions and to a lesser degree PTSD. While common among URMs, PTSD was rarely the cause of admission. Instead, underlying posttraumatic stress may have limited the capacity to cope with additional distress (Vujanovic et al. 2013). Co-morbidity was most likely common in the URM sample, but this was not analysed. The lack of diagnostic differences between URMs and non-URMs, except for cluster F40-48, is not unexpected. The literature indicating high morbidity among URMs is often based on self report instruments and non-clinical samples. Increased level of posttraumatic stress symptoms seem to be more consistent compared to depression (Harrington 1994; Thapar et al. 2012; Jakobsen et al. 2014). Further, need for inpatient treatment is not only related to diagnosis but also symptom severity. Finally, diagnostic assessments of refugee children may be a challenge due to lack of language and cultural skills in hospital staff and admitting physicians (Rousseau et al. 2008).

## Strengths

To our knowledge this is the first study analysing inpatient psychiatric treatment, including involuntary care, among URMs.

## Limitations

The number of URMs and their age span in the catchment area were based on an estimate. For precautionary reasons, the number of URMs was generously calculated, i.e. those that stayed only briefly in the catchment area are also included. Analysis on group level only is a limitation. Lack of analysis of specific diagnosis and co-morbidity is another constraint.

The delay in administering the questionnaire to the physicians may have affected the accuracy of the answers. However, the response rate was higher compared to other surveys to health professionals (Cook et al. 2009), which may indicate that URM-patients made a strong clinical impression and thus, to some degree, may have helped preserve the memory.

Due to the age difference in the URM and non-URM groups, a comparison of the sample divided according to developmental stage (12–14 and 15–17 years respectively) would be appropriate. However, in the age span 12–14 years there were only four URMs. This is too few to make a relevant statistical analysis. Further, the design of the study, including lack of access to individual medical records, prevents an analysis based on age 15 to 17 years. The study design and few URM-girls in the sample, does not allow a regression analysis, which otherwise may have clarified the relative importance of URM-status, gender and age for the involuntary care outcome.

## Future implications

Further research is required to validate the findings. The results suggest a need for adjustment of psychiatric care services for URMs. Professional training of staff working in supervised homes should be considered.

## Conclusion

From an epidemiological perspective, URMs were overrepresented in inpatient psychiatric care.

## Ethics

According to Swedish legalisation, ethical vetting is not required for developmental work. The study complies with the guidelines of the Committee of Ethics in Sweden and the identity of the patients in the files was unknown to the researchers. The search, carried out by the department’s’ patient and information security advisor, was performed on a group level. No medical files were opened.

## Abbreviations

EC: European Council
EU: European Union
GAD: General anxiety disorder
ICD: International classification of diseases
PTSD: Posttraumatic stress disorder
UNHCR: United Nations High Commissioner for Refugees
URM: Unaccompanied refugee minor
